# Synopsis of Barrier Function of Skin and Mucosa—Volume 2

**DOI:** 10.3390/ijms241813690

**Published:** 2023-09-05

**Authors:** Philip W. Wertz

**Affiliations:** Dows Institute for Dental Research, College of Dentistry, University of Iowa, Iowa City, IA 52242, USA; philip-wertz@uiowa.edu

This is an attempt to briefly summarize the contributions to this second Special Issue of the *International Journal of Molecular Sciences* on the barrier function of the skin and the oral mucosa.

The primary permeability barrier of the skin consists of the stratum corneum, in which flattened corneocytes with a monolayer of lipid covalently attached to the outer surface are surrounded by lipid lamellae, consisting of saturated fatty acids, cholesterol and ceramides [1]. In addition, the epidermis is protected from UV damage by a combination of melanin and urocanic acid. Protection against pathological microorganisms is provided by a variety of antimicrobial lipids, peptides and proteins and a low surface pH.

Tight junctions in the viable epidermis below the stratum corneum restrict the outward movement of water and water-soluble substances. A normal human stratum corneum also contains remnants or components of tight junctions fixed within the envelopes [2]. Electron microscopy has revealed tight junction remnants in the outer region of the stratum corneum of explant skin. These tight junction remnants are found at apical–lateral points of contact between adjacent corneocytes. When the barrier of the stratum corneum of reconstructed human epidermis was disrupted by treatment with caproyl salicylic acid for 24 h, an increase of tight junction remnants was found in the lowest two layers of the stratum corneum. It was proposed that tight junction components associated with these contact points may enhance cohesion of the stratum corneum and may reduce the speed of desquamation by limiting access to corneodesmosomes. Claudin is one component of tight junctions. In humans, claudin 1-negative mutants develop a syndromic ichthyosis. As part of this condition, tight junction remnant contact points persist throughout the stratum corneum even after corneodesmosomes have been degraded. This provides compensative reinforcement of the stratum corneum. Peeling skin disease results from mutations in the gene coding for corneodesmosin. The resulting fragility of the corneodesmosomes may be compensated for by upregulation of the tight-junction-derived envelope fusions.

The portion of the gingival epithelium adjacent to the tooth is the junctional epithelium. This junctional epithelium provides a tight junction barrier against the ingress of bacteria or bacterial products such as lipopolycaccharide [3]. Gram-negative bacteria predominate within the dental biofilm and produce lipopolysaccharide, a major driving force in the development of periodontitis. Lipopolysaccharide can cause disruption of the barrier of the junctional epithelium. Proteases produced by bacteria such as *Porphyromonas gingivalis* can also disrupt this barrier. Tight junction function decreases with advanced aging. Lipopolysaccharide can stimulate oral keratinocytes to generate a number of chemokines and cytokines, which then attracts neutrophiles into the sulcus. The production of oncostatin M by neutrophils can promote barrier dysfunction. The neutrophils themselves can mediate tissue damage by releasing proteases, cytokines and other factors. Mechanical rupture of the barrier is essential for the induction of bacteremia. This can result from mastication, tooth brushing or dental manipulations.

The human skin microbiota and its symbiotic relationship with the skin barrier has previously been examined [4]. The human microbiome refers to the collective genome of the microbiota and varies by anatomic site and life stage. The microbiota is essential for barrier function, at least partly through interaction with aryl hydrocarbon receptors on keratinocytes. *Staphylococcus epidermidis* secretes sphingomyelinase, which assists in the conversion of sphingomyelins to ceramides, which are important barrier components. This reaction also produces phosphorylcholine, which serves as a microbial nutrient source. Microbial interactions are also important for various aspects of innate and adaptive immunity. The natural microbiota also ward off invasion, colonization and infection by a variety of potential pathogens. The skin and gut have a bidirectional connection wherein alterations of the microbiome in one influence the other.

A carboxymethyl derivative of bacterial cellulose loaded with an extract of tumeric was characterized and appears to be a suitable candidate for wound dressing applications [5]. The composite demonstrates good absorbency, which is an important characteristic of a wound dressing with regard to exudate adsorption. It releases curcumin at a reasonable rate and has demonstrated antimicrobial activity against *Escherichia coli*, *Staphylococcus aureus* and *Candida albicans* while being nontoxic toward L929 fibroblasts.

Lithospermic acid (LA) is a phenolic compound that can be extracted from *Salvia miltiorrhiza*, also known as Danshen or red sage. Extracts of this plant have been used in traditional Chinese medicine to treat heart disease, high blood pressure and skin wounds, among other disorders. Lithospermic acid has also been shown to restore barrier function in an animal model of psoriasis [6]. Since LA has a molecular weight greater than 500 daltons, it must be formulated in an oil–water microemulsion containing surfactants in order for it to penetrate across the stratum corneum. In a study on psoriasis-like dermatitis induced via imiquimod in BALB/c mice (producing measurable changes in skin morphology, histology, transepidermal water loss, hydration and erythema), a topical LA formulation was able to significantly reduce all imiquimod-induced changes. The cytokines of the IL-23/IL-17 axis were downregulated via LA treatment.

The three review articles and one original research article in this Special Issue cover the discrete yet interrelated aspects of the barrier function of the skin and the oral mucosa [1,2,3,4]. The primary barrier function of the skin is provided by the stratum corneum, which contains an unusual array of lipids in the spaces between cornified cells [1]. However, in the viable epidermis beneath the skin’s stratum corneum, there are tight junctions that limit the outward movement of water and water-soluble substances [2]. When the barrier provided by the stratum corneum of skin is compromised, there is a measurable increase in tight junction components found in the stratum corneum. These tight junction components become cross-linked into the corneocyte envelopes and can be seen at points of fusion between adjacent corneocytes. This leads to increased cohesion of the stratum corneum and decelerates desquamation. There are similar tight junctions below the stratum corneum in the gingival epithelium, but in the sulcus, the junctional epithelium is a nonkeratinizing epithelium that attaches to the tooth surface [3]. Tight junctions in the junctional epithelium provide a barrier to the ingress of oral pathogens and the development of periodontal disease. The tight junctions can be compromised by the production of lipopolysaccharide by oral pathogens and by the production of oncostatin by neutrophiles. The bacteremia resulting from a breach of the gingival barrier is often a transient event following physical stress from mastication, tooth brushing or dental manipulations. Ongoing efforts to minimize periodontal disease include the use of probiotics to establish a healthier microbiome. Just as the production of lipopolysaccharide by Gram-negative oral pathogens contributes to breakage of the buccal barrier, microbiome dysbiosis can adversely affect the barrier function of the skin [4]. That the low pH of the skin’s surface and the presence of sebaceous lipids can serve as carbon sources for some bacteria, while being antimicrobial for others, are among the factors determining the health of the skin microbiome [1].
